# Imaging of Hidradenitis Suppurativa and Its Complications

**DOI:** 10.1155/2014/294753

**Published:** 2014-08-21

**Authors:** Feng Poh, Siew Kune Wong

**Affiliations:** Department of Diagnostic Radiology, Singapore General Hospital, Outram Road, Singapore 169608

## Abstract

We present a 56-year-old man with known diabetes mellitus and a 10-year history of hidradenitis suppurativa (HS) and highlight, through MR imaging findings, the relentless natural progression of the disease, characterized by recurrent exacerbations of abscesses and fistulae and complications of perianal fistulae and sacral osteomyelitis. We also demonstrate the appearance of this condition on PET-CT with F-18 FDG, which was performed for staging after postexcision tissue specimen revealed well-differentiated squamous cell carcinoma. The association of arthritis and possible dactylitis was also manifested in our patient. Discussion of HS in the radiology literature is limited and, to our knowledge, there has been no case report describing these imaging findings in the same patient.

## 1. Introduction

Hidradenitis suppurativa (HS) is a chronic debilitating cutaneous disorder characterized by recurrent development of inflammatory subcutaneous lesions, abscesses, fistulating sinus tracts, and eventual cicatrization. Uncommonly encountered by the radiologists due to the diagnosis being based largely on clinical assessment, HS is more than just “recurrent abscesses” and has several important associations such as Crohn's disease and arthritis that may warrant further evaluation as well as potential complications, including complex draining sinus and fistula tract formation, fistula-in-ano, lumbosacral epidural abscess from deep extension, osteomyelitis, and squamous cell carcinoma developing in chronic inflammatory tracts. Imaging plays a supplementary but important role in both the early and chronic stages of the disease, aiding in the characterization of active lesions, assessment of the extent of involvement, and the recognition of complications.

The use of advanced imaging modalities such as MRI, the modality of choice, has greatly aided in the management of these patients. To our knowledge, there has been limited discussion of HS in the radiology literature. We present a case report of a 56-year-old man with known diabetes mellitus and a 10-year history of HS and highlight through MR imaging findings, the relentless natural progression of the disease, characterized by recurrent exacerbations of abscesses and fistulae and complications of perianal fistulae and sacral osteomyelitis. We also demonstrate the appearance of this condition on PET-CT with F-18 FDG, which was performed for staging after postexcision tissue specimen revealed well-differentiated squamous cell carcinoma. The association of arthritis and possible dactylitis, which were also manifested in our patient, will also be discussed.

## 2. Case Report

A 56-year old man presents to our institution with complaints of increasing right buttock pain and pustular skin lesions, which have worsened in the recent month. Medical records showed recurrent admissions over the past 9 years for gluteal-femoral and perianal abscesses and multiple perianal fistulae requiring repeated surgical debridement. He has had gradual but certain weight loss in the recent years.

On clinical examination, extensive erythematous and pustular lesions were noted in the gluteal-femoral region bilaterally, with extension to the upper thighs (worse on the right), on background of rope-like dermal thickening and induration. Multiple fistulous openings were identified with visible purulent and haemoserous discharge. Perianal erythema is observed.

The patient was admitted for multidisciplinary management under colorectal surgery after being identified as a long-term sufferer of hidradenitis suppurativa (HS).

**Figure 1:**
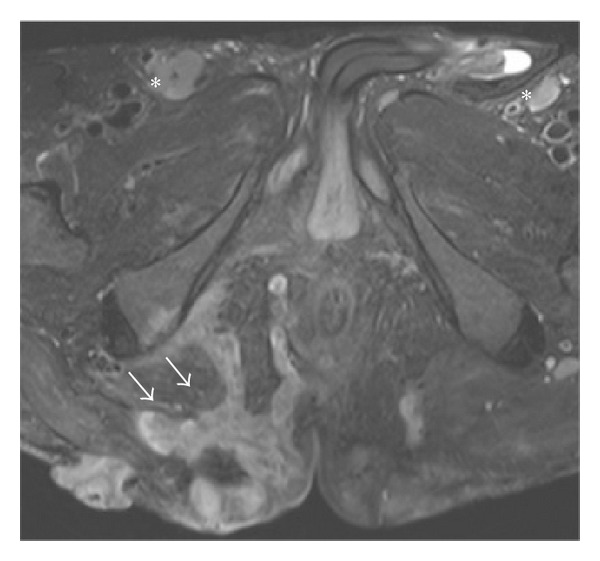
Axial FSE T2-weighted fat-suppressed image at presentation shows complex right gluteal subcutaneous collections with heterogeneous high T2-weighted signal intensity and low T1-weighted signal intensity (not shown). The tubular collections (white arrows) are reminiscent of branching primary and secondary sinus tracts and probably represent the chronic phase of these suppurative inflammatory tracts, extending to the deep right ischiorectal and ischioanal spaces. Bilateral dermal thickening and induration are seen. Bilateral enlarged inguinal lymph nodes are also demonstrated (∗).

**Figure 2:**
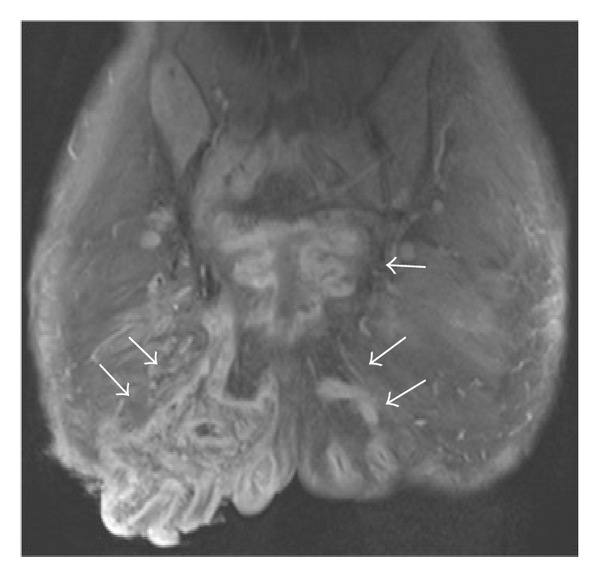
Coronal FSE T1-weighted fat-suppressed post-IV Magnevist 10 mls. Note: rim-enhancement of these collections post-IV contrast (white arrows), consistent with abscesses.

**Figure 3:**
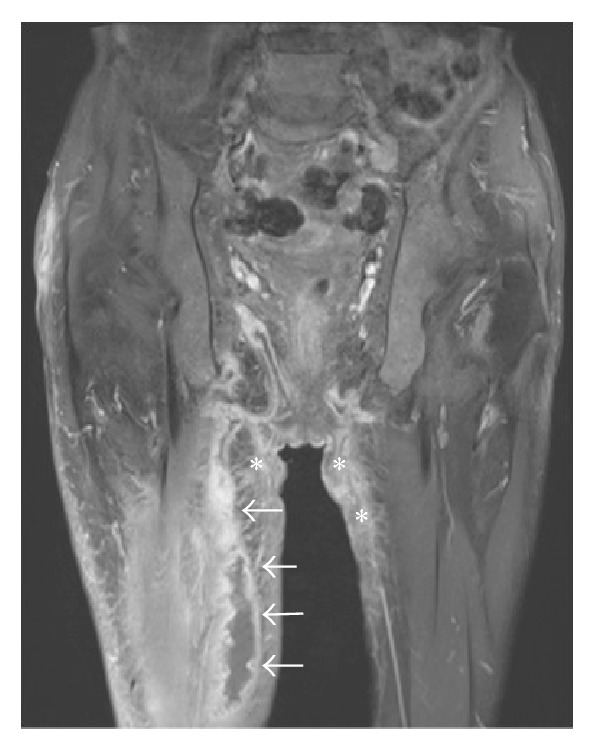
Coronal T1-weighted fat-suppressed image postcontrast shows inferior extension of the rim-enhancing abscess along the medial aspect of the right thigh (white arrows). Note: associated bilateral dermal induration best seen in the perineum with abnormal enhancement (∗).

An MRI of the pelvis was performed, which showed extensive subcutaneous abscesses, sinus, and fistula tracts in the right gluteal region where communicating and noncommunicating rim-enhancing collections were seen after IV contrast, extending deep into the right ischioanal fossa (Figures 1 and 2) and caudally to the right thigh (Figure 3). Marked skin thickening and subcutaneous induration over the lower back and both posterior thighs were observed.

**Figure 4:**
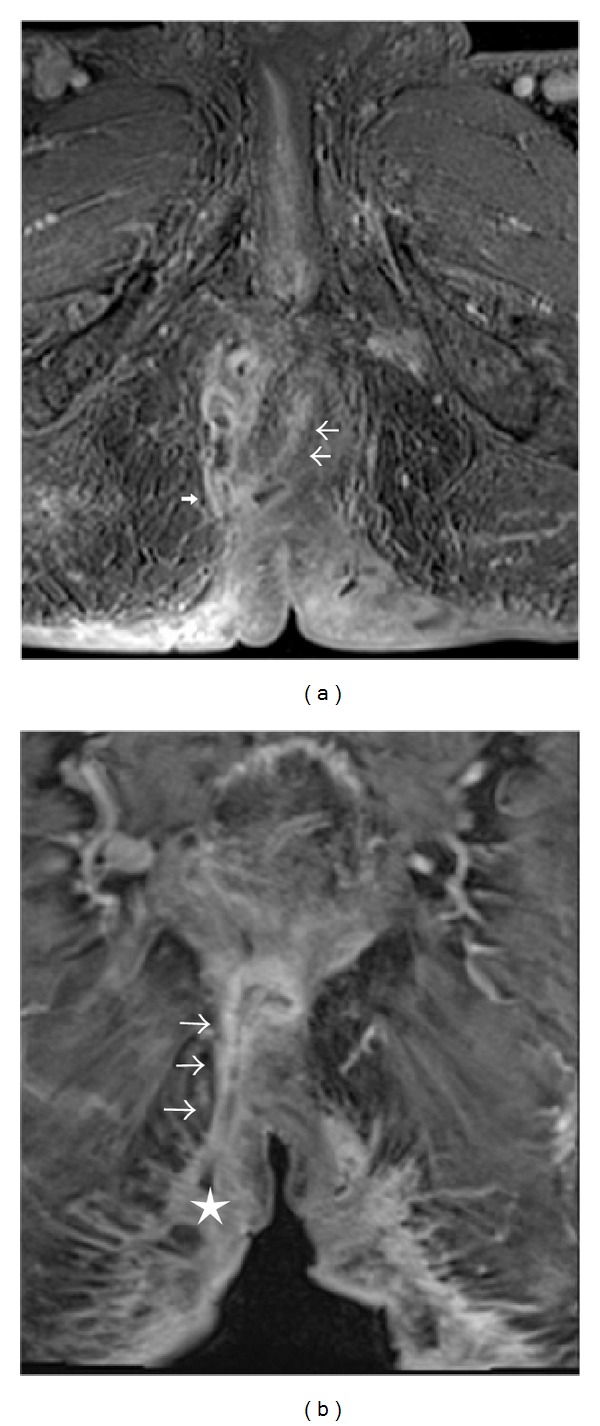
Axial T1-weighted fat-suppressed axial (a) and coronal (b) images postcontrast in an MRI pelvis done 3 years before presentation using fistula-in-ano protocol demonstrates an enhancing transphincteric fistula tract at 6 o'clock position (white arrows in both images) extending to the right ischioanal space (thick arrow) with cutaneous opening at the right gluteal region (star).

**Figure 5:**
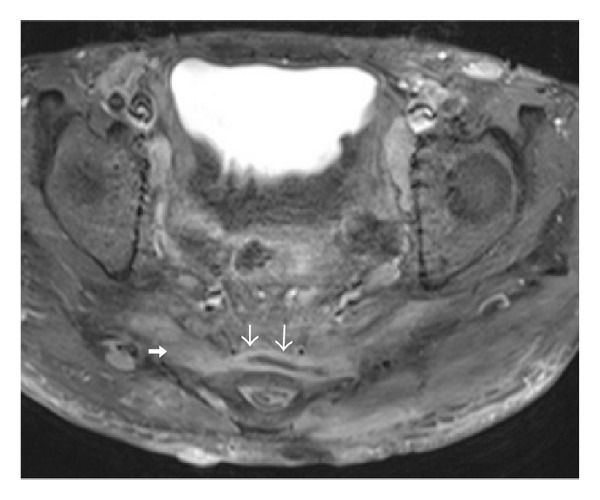
Axial T1-weighted fat-suppressed image postcontrast shows a well-defined rim-enhancing collection (white arrows) at the anterior perisacral space compatible with abscess, as well as abnormal marrow enhancement (thick arrow) in the sacrum consistent with osteomyelitis.

Similar to prior presentations, the deep right ischioanal abscess is closely related to a chronic transphincteric perianal fistula, better shown on a previous MRI (performed with fistula-in-ano protocol with smaller FOV) done 2 years before the current admission (Figure 4). There is also extension of the abscess to the anterior perisacral region, with adjacent sacral bony erosions and abnormal marrow enhancement indicating osteomyelitis (Figure 5).

Biochemical tests were consistent with sepsis and chronic inflammation, with raised total white count, polyclonal gammopathy and elevated erythrocyte sedimentation rate (ESR), and C-reactive protein (CRP) levels. Anemia, attributed to chronic disease, was also present. Initial wound culture grew* Proteus mirabilis* sensitive to Augmentin, with negative blood culture. A peripheral intravenous central catheter (PICC) was inserted and the patient was started on IV Augmentin, which was subsequently converted to IV vancomycin.

**Figure 6:**
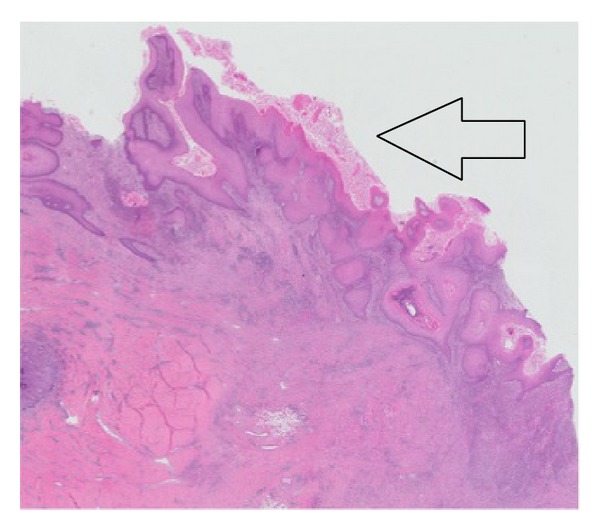
Tissue specimen of debrided right thigh shows skin surface lined by squamous cell carcinoma (open arrow).

He also underwent wide debridement and drainage of the multiple subcutaneous abscesses with discharging sinuses affecting bilateral gluteofemoral and pararectal regions. Tissue specimens that were sent for histology incidentally revealed well-differentiated squamous cell carcinoma that has arisen from the chronic sinus tracts at the right thigh (Figure 6).

**Figure 7:**
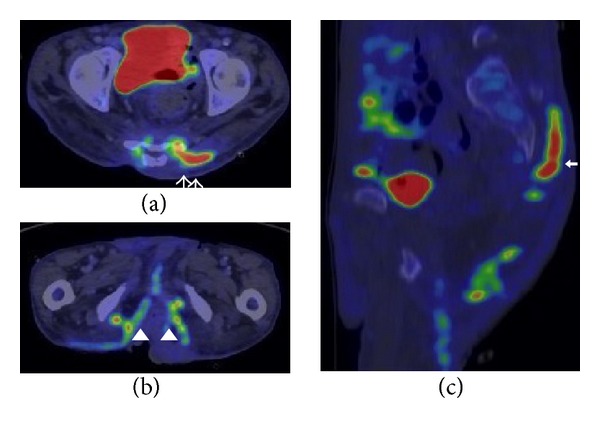
PET-CT performed after surgical excision of the right squamous cell carcinoma in the right thigh with wide resection margins. Note: FDG-avid foci in the perianal spaces ((b), arrowheads) and a more superior abscess collection at the left posterior back ((c), thick arrow), extending to the sacral level ((a), white arrows).

A PET-CT scan following IV F-18 FDG was performed for staging, which showed no evidence of focal hypermetabolic lesion to suggest distant metastases. FDG-avid inflammation was however observed at the lesions of HS consistent with the subcutaneous inflammation (Figure 7).

**Figure 8:**
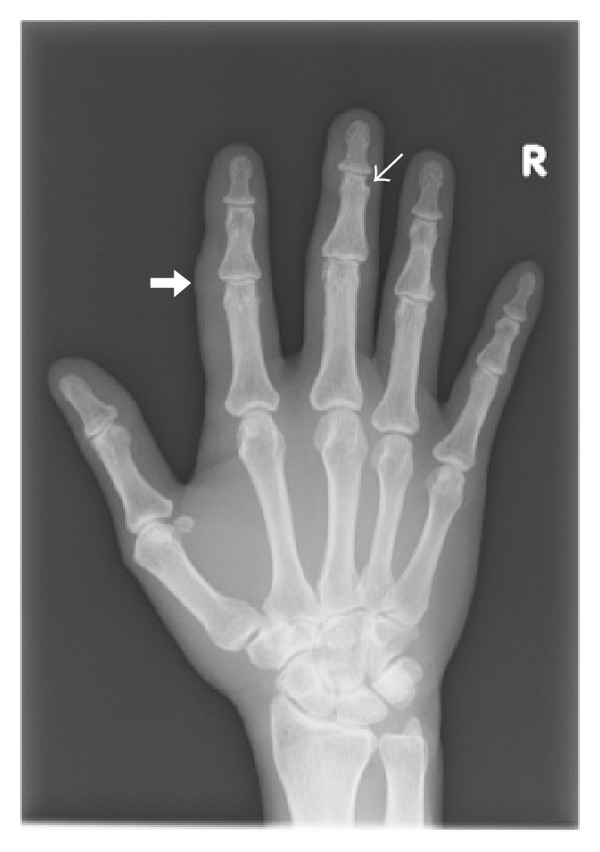
Frontal radiograph of the right hand shows focal periarticular soft tissue swelling at the proximal interphalangeal joint of the 2nd digit (thick arrow), suspicious for dactylitis. A small juxta-articular erosion is also noted at the middle phalanx of the middle finger (white arrow), which may represent the arthropathic changes related to HS.

During this admission, the patient also developed painful swelling of the 2nd digit of the right hand. Radiographs demonstrated periarticular soft tissue swelling at the 2nd proximal interphalangeal joint and a small juxta-articular erosion at the 3rd distal interphalangeal joint (Figure 8). These appearances, although nonspecific, may be related to HS-associated arthritis in the absence of previous history of inflammatory arthropathy.

Our patient underwent sigmoid loop colostomy for faecal diversion after debridement of the chronic perianal fistulae. He was also started on palliative radiotherapy and chemotherapy for treatment of the right thigh squamous cell carcinoma.

## 3. Discussion

First described by Velpeau in 1839 with subsequent work done by Verneuil, Schiefferdecker, Brunsting, and Pillsbury et al. [1–5], hidradenitis suppurativa has been well described as a chronic disabling disorder affecting the terminal follicular epithelium in the apocrine gland-bearing skin and is characterized by acneiform follicular occlusion and inflammation, relapsing mucopurulent infections, and progressive scarring and sinus formation.

Diagnosis is primarily clinical with recognition of typical lesions in characteristic distribution and recognizing its recurrent nature. Tender erythematous skin lesions are often identified on clinical examination during active inflammation, on background of dermal contractures and scarring from episodic recurrences. The anogenital regions and axillae show predilection for disease.

Known associations have also been described, with Plewig and Kligman introducing the concept of acne tetrad, including acne conglobata, dissecting cellulitis and pilonidal abscess and suggesting acne inversa as a more accurate name, reflecting the pathogenetic link to follicular occlusion [6, 7]. Other reported disease entities with possible associations with hidradenitis suppurativa are Crohn's disease and arthritis [8].

**Figure 9:**
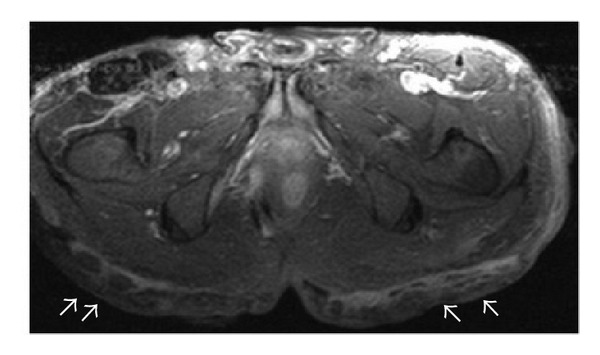
Axial T1-weighted fat-suppressed image postintravenous Magnevist: this MRI image done at the patient's first presentation 9 years ago shows multiple rim-enhancing subcutaneous abscesses in bilateral gluteal regions (white arrows) without deep extension or significant dermal induration, consistent with the early course of the disease.

As is commonly described, our patient presents at middle age with recurrent perianal abscesses and has since progressed from his initial presentation a decade ago, where there were isolated abscess formation (early stage, Figure 9) without dermal scarring or sinus formation through recurrent episodes of abscess and sinus tract formation with development of cicatrization and complex sinus tract formation. No evidence of Crohn's disease was found on previous colonoscopy and tissue biopsy in our patient, which is associated with recurrent fistula-in-ano.

Known to have a variable clinical course, the hallmark of HS is the development of sinus tracts, abscesses, and fistula formation, which were described by Nadgir et al. on double-contrast barium enema examination [9]. This clinical course may be influenced by predilection to bacterial infections, such as underlying diabetes mellitus, as in our patient, who developed extensive perisacral abscess and sacral osteomyelitis, not unlike the case report of lumbosacral epidural abscess secondary to HS described by Russ and Castillo who suggested a possible association between diabetes mellitus and HS [10].

In our patient, MRI consistently demonstrated, both in the early stage of the disease (Figure 9) and in the chronic stage (Figures 1–4), findings of subcutaneous abscesses, which were of low signal on T1-weighted images and high signal on T2-weighted fat-suppressed images and which showed peripheral rim-enhancement after intravenous contrast administration, as described by Kelly and Cronin in 2005 [11]. The current MRI also demonstrated imaging characteristics of chronic disease described in the same paper, with marked thickening of the skin and induration of the subcutaneous tissues appearing low signal on T1-weighted images and high signal on T2-weighted fat-suppressed images, consistent with tissue oedema. Reactive inguinal lymph node enlargement was also observed.

These areas of subcutaneous inflammation were noted to be hypermetabolic on the PET-CT study (Figure 7) done for staging, corresponding to the findings by Simpson et al. whom described the PET features of HS [12].

Our patient also developed the complication of squamous cell carcinoma, a well-reported association [13] that, although rare, is potentially fatal and which requires a high index of suspicion and early tissue diagnosis for initiation of prompt surgical treatment.

Notably, the patient also had pain and swelling of his right index finger of several weeks duration, which were further evaluated with radiographs that demonstrated periarticular soft tissue swelling of her affected digit as well as incidental bony erosion at the 3rd middle phalanx. This may be related to arthritis and possibly dactylitis associated with HS, recently described by Fioravanti et al. [14]. Arthritis associated with HS is uncommon and typically affects the peripheral joints in an asymmetrical distribution. The pathogenesis of the arthritis remains unknown and has been reported to be seronegative for the presence of rheumatoid factor. Imaging findings are nonspecific and include asymmetrical erosions, soft tissue swelling, periosteal reaction, and periarticular osteoporosis, with a suggestion of possible dactylitis that may precede the onset of arthritis [14]. The importance of the findings is the possibility of future development of seronegative arthritis, which may require interval clinical monitoring.

## 4. Conclusion

In conclusion, we presented the typical MR and PET-CT imaging findings of HS and radiographic findings of HS-related arthritis and demonstrated several complications associated with the disease, including perianal fistula, sacral osteomyelitis, and squamous cell carcinoma, in our 56-year-old male patient who required dedicated wound management, antibiotics therapy, and aggressive surgical debridement. Apart from the radiological findings, our patient also had clinical manifestations of raised inflammatory markers, anemia of chronic disease, chronic weight loss, and polyclonal gammopathy, which were consistent with the chronic debilitating phase of HS. The imaging findings in our patient corroborated with the current radiological literature on HS (Kelly and Cronin, Simpson et al., Fioravanti et al.) in terms of MRI, PET-CT, and HS-related arthritis and highlight important complications that radiologists should be aware of such as bony involvement, that is, osteomyelitis, possible epidural abscess formation, perianal fistulae, and the rare instance of degeneration to squamous cell carcinoma.
